# Serious Bacterial Infections in Preterm Infants: Should Their Age Be “Corrected”?

**DOI:** 10.3390/jcm12093242

**Published:** 2023-05-01

**Authors:** Mohamad Hadhud, Itai Gross, Noa Hurvitz, Lea Ohana Sarna Cahan, Zivanit Ergaz, Giora Weiser, Noa Ofek Shlomai, Smadar Eventov Friedman, Saar Hashavya

**Affiliations:** 1 Department of Pediatrics, Hadassa—Hebrew University Medical Center, Hebrew University of Jerusalem, Jerusalem 91120, Israel; 2 Department of Pediatric Emergency Medicine, Hadassah—Hebrew University Medical Center, Faculty of Medicine, Hebrew University of Jerusalem, Jerusalem 91120, Israel; 3 Department of Pediatrics, Hadassah Medical Center, Ein Kerem, Kiryat Hadassah, POB 12000, Jerusalem 91120, Israel; 4 Department of Neonatology, Hadassah—Hebrew University Medical Center, Faculty of Medicine, Hebrew University of Jerusalem, Jerusalem 91120, Israel; 5 Department of Pediatric Emergency Medicine and Shaare Zedek Medical Center, Jerusalem 91120, Israel

**Keywords:** emergency medicine, premature infants, serious bacterial infection, corrected age, ex-premature infants

## Abstract

Adjusting the chronological age of preterm infants according to their gestational age is a widely accepted practice in the field of neurodevelopment. It has been suggested for the assessment of preterm infants with suspected infection, but has been poorly validated. Correcting for chronological age is especially critical in infants with a chronological age above 3 months, but a corrected age below 3 months due to the differences in assessment protocols. This study assessed the difference in incidence of serious bacterial infection (SBI) according to chronological and corrected age in preterm infants. A retrospective analysis of pediatric emergency department (PED) presentations was conducted for all 448 preterm infants born in between January 2010 and August 2019. Of the 448 preterm infants, 204 (46%) presented at one of 3 PEDs in Jerusalem, Israel, during their first year of life. Overall, 141 (31.4%) presented with fever and were included in the study. The infants were divided into 3 age groups: 1—corrected age >3 months; 2—chronological age >3 months, but corrected age <3 months; 3—chronological and corrected age <3 months. SBI was diagnosed in 2.6%, 16.7%, and 33.3% of the infants in groups 1, 2 and 3, respectively; (*p* < 0.01, *p* = 0.17, *p* < 0.001). The incidence of SBI in the control group of 300 term infants <3 months presenting to the PED due to fever was 15.3%. Preterm infants with a corrected age <3 months are at increased risk for SBI, similarly to term infants <3 months of age. Age correction should thus be considered for preterm infants presenting with fever.

## 1. Introduction

An estimated 15 million infants are born prematurely every year worldwide, at increasing survival rates, even when born at an early gestational age. However, prematurity is associated with increased postnatal morbidity, mostly in the first week of life, and ranks as the second leading cause of death globally in children under the age of five [1]. Prematurity is defined as childbirth occurring before 37 weeks of gestation [2] and is commonly classified into extremely preterm (less than 28 weeks gestational age), very preterm (28 to 32 weeks gestational age), and late preterm (32 to 37 weeks gestational age) [3]. The younger the gestational age at birth, the higher the risks of morbidity and mortality [4].

For health, growth, and developmental evaluations, it is widely accepted to adjust preterm gestational age. Adjusted age, also commonly known as corrected age, is calculated by subtracting the number of weeks that the child was born prematurely from the child’s chronological age, defined as a 40-week full-term pregnancy [5]. This assessment is generally applied until at least 24 months of age when monitoring premature infants’ growth and neurodevelopmental progress, including cognitive function, language, and motor and social interaction skills. It is used to eliminate the bias and decrease the transient developmental gap of prematurity until these infants catch up with their full-term peers [6,7].

Studies have shown that premature infants are more vulnerable to infections in the first year of life and, in particular, that the risk of severe infection and hospitalization is inversely proportional to gestational age and birth weight [8,9,10]. Besides perinatal comorbidities, preterm infants have an immature immune system, with an inadequate adaptive and innate immune response. Thus, an age correction is often suggested for the assessment of a premature infant with suspected infectious disease; however, this assumption has not been extensively validated [11].

Infants younger than 90 days who present with fever are traditionally considered to have increased vulnerability for infections and may only exhibit subtle clinical manifestations, even in the case of serious bacterial infection (SBI). For this reason, different assessment algorithms, such as the Philadelphia, Boston, Milwaukee, and Rochester criteria, have been applied to stratify the risk of SBI in this population of infants younger than 60 or 90 days [12,13,14]. Recent algorithms, such as the Mintegi, Kuppermann, and Aronson formulas, have emerged in the past few years [15,16,17,18]. In most of these assessment algorithms, preterm infants under 60 or 90 days are considered to have high risk for SBI. Infants, including preterm infants, above the chronological age of 60 or 90 days are not included in these algorithms at all. Preterm infants with a chronological age above the inclusion age in these algorithms, but a corrected age below it may thus be assessed differently depending on whether their corrected or chronological age is taken into account. For example, immunization schedules in many countries, including Israel, are based on chronological rather than corrected age.

Only a few studies have evaluated the incidence and management of preterm infants presenting at the pediatric emergency department (PED) in their first year of life [10,11]. Most have examined infants with a chronological age over 3 months. Studies investigating infants born at a younger gestational age are limited. Thus, the objective of this study was to evaluate the incidence of infectious episodes, including SBIs, in preterm infants after discharge from the neonatal intensive care unit (NICU), especially those with a chronological age above 3 months, but a corrected age below 3 months.

## 2. Materials and Methods

### 2.1. Sample

This retrospective study was conducted at the 3 major PEDs in Jerusalem, Israel, with 90,000 annual PED presentations. Premature infants born at our medical centers from January 2010 to August 2019 at a gestational age of 23 to 32 weeks who presented at one of these PEDs during their first year of life were included.

To be as comprehensive as possible, all infants with a suspected infection, as indicated by their chart reports (i.e., temperature equal to or above 38 °C or less than 35.5 °C; not feeding well; and displaying apathy, restlessness, vomiting, or dyspnea), were further evaluated and compared as a function of their chronological and corrected age. Infants who presented at the PED for non-infectious causes were not further evaluated. Infants with missing medical records were excluded from the study. A control group composed of 300 term infants below the age of 3 months who presented at the PED with suspected infections was included. The charts of age-matched term infants were retrieved randomly.

SBI was defined as at least one of the following diagnoses: bacterial meningitis (positive cerebrospinal fluid culture (CSF)); bacteremia (the presence of a positive blood culture); or urinary tract infection (UTI), with a positive urine culture defined as growth of a single organism ≥ 100,000 colony forming units/mL (CFU/mL) for clean catch, ≥10,000 CFU/mL for a catheterized specimen, or ≥1000 CFU/mL in urine collected by suprapubic aspiration (SPA). For both CSF and blood cultures, positive culture results were classified as true pathogens or contaminant species based on the attending physician’s treatment plan, but only pathogenic bacteria were included in the analysis of the positive blood and CSF culture findings. Positive urinalysis was defined as positive leukocyte esterase or nitrite on urine dipstick. CSF pleocytosis was defined as an increased nucleated cell count >5 cells/µL. Chronological age was defined as the age calculated from the day of birth. Gestational age was defined as the time elapsed between the first day of the last normal menstrual period and the day of delivery. Corrected age in months was defined by subtracting [40 weeks minus the gestational age (weeks)]/4 from the chronological age in months [10]. Based on this calculation, the cohort of preterm infants was classified into 3 groups: Group 1: Infants with a chronological and corrected age over 3 months; Group 2: Infants with a chronological age over 3 months and corrected age below 3 months; Group 3: Infants with both a chronological and corrected age of less than 3 months. The control group was composed of term infants below the age of 3 months.

The late preterm group (preterm older than 32 weeks) was excluded from analysis since our main interest was in the younger preterm groups, a population that is more likely to be affected by age correction protocols since the gap between the corrected and chronological age is the greatest.

### 2.2. Data Collection

IRB approval for this study was secured from all the participating medical centers. The data were collected retrospectively from medical records. Infants were evaluated for gestational age; birth weight (BW) and gender; NICU course, including respiratory support and other treatment; bronchopulmonary dysplasia (BPD); necrotizing enterocolitis (NEC); Intraventricular hemorrhage (IVH); and age at discharge from the NICU.

The computerized data for infants presenting at the PED were evaluated for the reason for the visit, age, and clinical signs upon admission. Ancillary laboratory test results, including inflammatory markers and microbiological tests results (urine, blood, and CSF), chest X-ray findings, antibiotic treatment, and hospitalization course (including Pediatric Intensive Care Unit (PICU) admission, length of stay (LOS), complications, and mortality), were collected.

### 2.3. Statistical Analysis

The statistical analysis was conducted using SPSS version 21.0 (Statistical Package for Social Science, Chicago, IL, USA). Continuous variables are presented as the mean ± SD or the median and interquartile range. The categorical variables are expressed as frequencies and percentages. A chi-square test was used to compare proportions, and a Student *t*-test was used to compare continuous parametric variables. A *p* value of ≤0.05 was considered statistically significant.

## 3. Results

**Figure 1:**
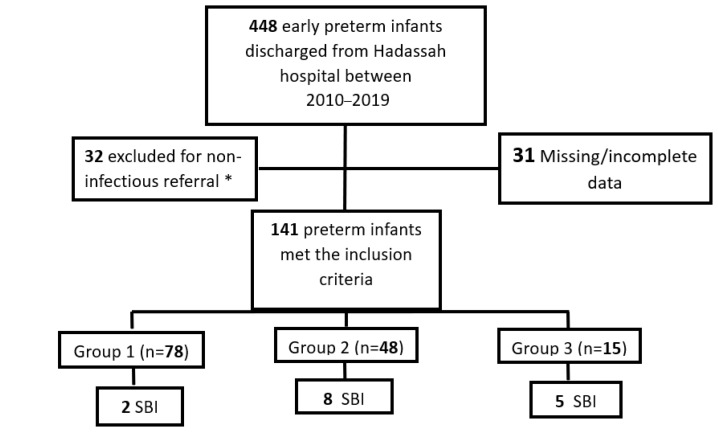
Infants in the second and third groups had a significantly longer LOS than infants in the first group: 5.43 ± 10 days, 8.7 ± 6.7 days, and 2.06 ± 4.1 days, respectively (*p* = 0.01, *p* < 0.0001). The rate of admission to the PICU was significantly higher in the third (13.33%) than in the first group (1.28%) (*p* = 0.01). There was no statistical difference between the third (13.33%) and the second group (14.58%) (*p* = 0.94) (Table 1). Patient selection flowchart. Group 1: Infants with a chronological and corrected age >3 months. Group 2 Infants with a chronological age >3 months but a corrected age <3 months}. Group 3: Infants with both a chronological and corrected age <3 months. * Excluded for non infectious (e.g., hernia, PEG replacementfractures and head trauma), PEG: Percutaneous endoscopic aastrostomy, SBI: serious bacterial infection.

Of the 448 eligible preterm infants born at our hospital between 2010 and 2019, 204 (46%) presented at 1 of the 3 PEDs during their first year of life. Of these, 32 had a non-infectious diagnosis, and 31 were excluded due to missing data. Of the 141 patients who met the inclusion criteria, 78 (55.3%) were classified as group 1, 48 (34%) as group 2, and 15 (10.6%) as group 3 (Figure 1). The male–female ratio was 2.06 (95/46), with a mean age at presentation of 115.9 days (SD ± 94.7), a mean gestation age of 27.9 weeks (SD ± 1.8), and a mean age at discharge from the NICU of 80 days (SD ± 41).

Of the 141 infants with suspected infection, fever was the most common cause for referral to the PED and was reported in 66 (46.8%) infants. Fever was more common in group 1 than in groups 2 and 3 (*p* = 0.003 and *p* = 0.0002, respectively) (Table 2).

SBIs accounted for 10.6% (n = 15) of all cases, of which 2 (2.56%) were in group 1, 8 (16.66%) in group 2, and 5 (33.33%) in group 3, (Supplemental Table S1). Infants in groups 2 and 3 had a significantly higher incidence of SBI than in group 1 (*p* <0.01 and *p* < 0.001, respectively), whereas no difference was found between groups 2 and 3 in terms of SBI (*p* = 0.17).

A total of 15 infants had 20 cases of SBI (3 infants were diagnosed with UTI and 2 infants with meningitis had concomitant bacteremia). UTI was the most common SBI in all age groups, as well as in the control group, and was significantly more common in groups 2 and 3 than in group 1 (*p* = 0.01 and *p* = 0.03, respectively) (Supplemental Table S1). *Escherichia coli* was the most common pathogen found in 11/20 (55%) of all cultures (CSF, blood, and urine), followed by *Streptococcus pyogenes* 3/20 (15%) and *Proteus mirabilis* 2/20 (10%). Four blood cultures with contaminants (Streptococcus salivarius, Streptococcus mitis, staphylococcus epidermidis, streptococcus intermedius) were excluded from the analysis. All cases of bacterial meningitis had concomitant bacteremia and occurred in group 2 (*p* = 0.07). Of these, one had concomitant ventriculitis and required prolonged PICU admission.

The incidence of SBI in the control group was 46/300 (15.3%), similar to group 2 (*p* = 0.8), but significantly higher (*p* = 0.003) than in group 1. More cases of SBI were found in the third group 5/15 (33%) than in the control “term infants” group 46/300 (15.3%), although the difference did not reach statistical significance (*p* = 0.06), (Table 3).

The incidence of complications during the primary hospitalization in the NICU did not differ across age groups. The typical complications of prematurity are listed in Supplemental Table S2. Infants with SBI had an increased rate 14/15 (93.3 %) of a complicated course of treatment during their primary hospitalization in the NICU (Supplemental Table S3).

Ancillary laboratory tests of the infants with SBI did not differ across age groups (Supplemental Table S4). All cases of bacteremia and meningitis had white blood cell abnormalities (WBC above 15 × 10^3^ or under 5 × 10^3^) and elevated C-reactive protein (CRP > 2.5). All the patients with meningitis had cerebrospinal pleocytosis.

In total, 126 infants had an infectious episode that was not SBI. Bronchiolitis was the most common diagnosis in 55 (39%), followed by other viral infections in 42 (29.8%), of whom 2 cases were diagnosed as viral meningitis. This was followed by upper respiratory tract infection (URTI) (7.8%), acute otitis media (5%), gastroenteritis (2.8%), cellulitis (2.1%) pneumonia (1.4%), and pertussis (1.4%). There was no statistical difference between groups in terms of non-SBI diagnoses (Table 4).

In total, 5 (9%) of the infants diagnosed with bronchiolitis had a severe course and required mechanical ventilation, 3 (60%) of whom were from group 2, and 1 (20%) from groups 1 and 3 each. Two suffered from BPD. No mortality was recorded.

## 4. Discussion

In this study, preterm infants with a corrected age of less than 3 months had an increased incidence of SBI, similar to term infants below the age of 3 months. The correction for gestational age, a practice used in other areas of pediatrics, is currently employed primarily for the assessment of child development, but is not routinely taken into account when dealing with SBI in preterm infants presenting at the PED, although it is often used by pediatricians [11].

Numerous studies have reported an incidence of SBI ranging from 5% to 15% in term infants below the age of 3 months. Preterm infants constitute a special subgroup of infants below the age of 3 months, and most protocols consider them a priori to be a high-risk group that requires prompt antibiotic treatment, despite the small number of studies on the incidence of SBI in preterm infants [12,13,14]. There is little to no literature on the assessment and risk stratification of SBI for preterm infants, especially those with a chronological age above 3 months, but a corrected age below 3 months, and these infants tend not to be included in reported protocols.

For example, a retrospective study conducted in California found an increased hospitalization rate for early preterm infants when compared to late preterm and term infants in the first year of life, which was also the case for the first 90 days of life [8]. Another study showed that rates of infection-related hospitalization were higher for preterm infants and were inversely correlated with gestational age [9]. However, none of these studies addressed the need for age correction in preterm infants, and to the best of our knowledge, only one study has dealt with the need for age correction in the PED setting. That study assessed the PED presentation of late preterm infants with a postconceptional age < 48 weeks in a single PED, and found that preterm infants with a corrected age < 8 weeks had a SBI rate (9.2%) that was similar to the published SBI rate for term infants [11].

In the current study, preterm infants with a corrected age of less than 3 months and a chronological age of more than 3 months (group 2) had a similar incidence of SBI as reported in term infants below the age of 3 months [19,20,21], thus further emphasizing the similarities between the 2 groups and supporting age correction. The incidence of SBI in preterm infants with a corrected age > 3 months (group 1) was similar to the reported incidence of 1.8% to 4.2% in term infants aged 3 to 36 months [22,23]. In these two groups, age correction was found to correlate better in terms of SBI incidence than chronological age.

Preterm infants with a chronological age of less than 3 months (group 3) were especially vulnerable to SBI, with the highest incidence of all groups including the control group 5/15 (33.3%), thus emphasizing the need for a high index of suspicion and prompt antibiotic treatment.

As reported elsewhere, UTI was the most common SBI in all groups [10,14,24]. *Escherichia coli* was the most common pathogen in all groups, as was previously reported for term infants below the age of 90 days [25]. In the current study, the complete blood count, urinalysis, and CRP levels were of limited value in order to predict SBI, similar to what has been reported in term infants younger than 90 days [25,26].

Among the non-SBI diagnoses, bronchiolitis was the leading cause for presentation at the PED in all groups, followed by non-specified viral infections, which is in line with studies in both term and preterm infants [27].

This study has several limitations. First, due to its retrospective nature, the findings were limited by the availability of information from patients’ charts. Secondly, it is possible that some of the infants presented to other medical centers in Israel. To minimize this issue, referrals to the three major PEDs in the metropolitan area were assessed. We believe this measure significantly reduced the possibility of unreported SBI admissions. Finally, the cohort was somewhat small, so the statistical analysis was limited. However, the study included a variety of preterm infants born in our medical center over a decade and covered the busiest PEDs in Jerusalem.

## 5. Conclusions

This study highlights the differences in characteristics and medical complications of premature infants, as compared to term controls, and their impact on the increased incidence of SBI in these infants. The findings support the claim that an age correction of preterm infants should be considered since the risk of SBI correlates with the corrected rather than the chronological age. Preterm infants with a chronological age of less than 3 months emerged here as especially vulnerable to SBI as compared to term infants. We recommend that premature infants, especially those below a corrected age of 3 months, should be evaluated with caution and considered for more extensive care when presenting at the PED.

## Figures and Tables

**Table 1:** Demographics.

	All Patients (n = 141)	Group 1 (n = 78)	Group 2 (n = 48)	Group 3 (n = 15)	Group 1 vs. Group 2 *p* Value	Group 2 vs. Group 3 *p* Value	Group 1 vs. Group 3 *p* Value
Male (%)	95 (67.4%)	53 (67.9%)	32(66.7%)	10 (66.7%)	0.87	1	0.92
Gestational age (weeks)	27.9 ± 1.8	27.9 ± 1.9	27.8 ± 1.7	28.6 ± 1.2	0.82	0.3	0.31
Birth weight (grams)	1104 ± 297.8	1089 ± 314	1118 ± 281.1	1139 ± 272.5	0.79	0.98	0.72
Corrected age at presentation to ED (days)	115 ± 94.7	185.7 ± 67	41 ± 23	−7.9 ± 10.9	<0.0001	<0.0001	<0.0001
Chronological age at presentation to ED (days)	115.9 ± 94.7	185.8 ± 67	125.8 ± 26.1	71.5 ± 1.8	<0.0001	<0.0001	<0.0001
Length of stay in hospital (days)	3.922 ± 7.28	2.06 ± 4.1	5.43 ± 10	8.7 ± 6.7	0.01	0.32	<0.0001
Antibiotic administration (%)	46 (32.62%)	20 (25.64%)	16 (33.33%)	10 (66.7%)	0.37	0.02	0.001
PICU admission (%)	10 (7.09%)	1 (1.28%)	7 (14.58%)	2 (13.33%)	0.18	0.94	0.01

**Table 2:** Reasons for referral to the emergency department.

	All Patients	Group 1	Group 2	Group 3	Group 1 vs. Group 2	Group 2 vs. Group 3	Group 1 vs. Group 3
	(n = 141)	(n = 78)	(n = 48)	(n = 15)	*p* Value	*p* Value	*p* Value
Fever	66 (46.8 %)	47 (60.25%)	16 (33.3%)	3 (6.7%)	0.003	0.04	0.0002
Dyspnea	47 (33.3%)	20 (25.64%)	19 (39.6%)	6 (40%)	0.09	0.98	0.23
Restlessness	7 (5%)	1 (1.28%)	6 (12.5%)	0	0.007	0.15	0.66
Apathy	10 (7.1%)	4 (5.12%)	5 (10.4%)	1 (6.7%)	0.26	0.67	0.75
Vomiting	10 (7.1%)	6 (7.69%)	2 (4.2%)	2 (13.3%)	0.43	0.21	0.48
Swelling of the eyes	1 (0.7%)	0	0	1 (6.7%)	1	0.49	0.38

**Table 3:** Comparison of serious bacterial infections across groups vs. the control group of term infants.

	Group 1 (n = 78)	Group 2 (n = 48)	Group 3 (n = 15)	Control (n = 300)	Control vs. Group 1 *p* Value	Control vs. Group 2 *p* Value	Control vs. Group 3 *p* Value
**Serious bacterial infection, total (%)**	2 (2.6%)	10 (20.8%)	5 (33.3%)	46 (15.3%)	0.003	0.34	0.06
**Meningitis (%)**	0	2 (4.16%)	0	3 (1%)	0.38	0.09	0.69
**Bacteremia (%)**	0	4 (8.3%)	2 (13.3%)	8 (2.67%)	0.14	0.05	0.02
**Urosepsis (%)**	0	2 (4.16%)	1 (6.7%)	4 (1.33%)	0.3	0.16	0.1
**Urinary tract infection (%)**	2 (2.6%)	6 (12.5%)	4 (26.7%)	39 (13%)	0.009	0.92	0.13
**Age at presentation in days (SD)**	270.3 (66.9)	127.4 (27.9)	71.5 (12.4)	45.2 (22.4)	*p* < 0.0001	*p* < 0.0001	*p* < 0.0001
**Corrected age, days (SD)**	185.8 (67)	42 (24.6)	7.8 (10.5)	45.2 (22.4)	*p* < 0.0001	0.37	*p* < 0.0001
**Gender, males (%)**	53 (67.9%)	32 (66.7%)	10 (66.7%)	174 (58%)	0.11	0.26	0.5

**Table 4:** Comparison of non-SBI infections across groups.

Diagnosis	All patients (n = 141)	Group 1 (n = 78)	Group 2 (n = 48)	Group 3 (n = 15)	Group 1 vs. Group 2 *p* Value	Group 2 vs. Group 3 *p* Value	Group 1 vs. Group 3 *p* Value
**Bronchiolitis**	55 (39%)	33 (42.3%)	19 (39.58%)	3 (20%)	0.11	0.16	0.83
**Upper respiratory tract infection**	11 (7.8%)	7 (8.97%)	3 (6.25%)	1 (6.7%)	0.8	0.96	0.54
**Acute otitis media**	7 (5.0%)	6 (7.69%)	1 (2.08%)	0	0.26	0.58	0.17
**Pertussis**	2 (1.4%)	0	1 (2.08%)	1 (6.7%)	0.02	0.36	0.21
**Gastroenteritis**	4 (2.8%)	3 (3.84%)	1 (2.08%)	0	0.43	0.58	0.57
**Pneumonia**	2 (1.41%)	2 (2.56%)	0	0	0.54	1	0.26
**Skin infection**	3 (2.1%)	1 (1.28%)	1 (2.08%)	1 (6.7%)	0.19	0.58	0.76
**Viral infection**	42 (29.78%)	24 (30.76%)	14 (29.16%)	4 (26.7%)	0.76	0.86	0.83

## Data Availability

Data is available following a written request to itaig@hadassah.org.il.

## Supplementary Materials

The following supporting information can be downloaded at: https://www.mdpi.com/article/10.3390/jcm12093242/s1, Table S1: Rate of serious bacterial infection in the different preterm age groups; Table S2: Postnatal complications during NICU admission; Table S3: Postnatal complications in preterm infants with SBI during NICU admission; Table S4: Ancillary laboratory test results in preterm infants with SBI.

## Abbreviations

SBI—Serious bacterial infection, PED—Pediatric emergency department, NICU—Neonatal intensive care unit, PICU—Pediatric intensive care unit, CSF—Cerebrospinal fluid culture, UTI—Urinary tract infection, BW—Birth weight, BPD—Broncho pulmonary dysplasia, NEC—Necrotizing enter-colitis, IVH—Interventricular hemorrhage, LOS—length of stay, CRP—C-reactive protein, URTI—Upper respiratory tract infection.
