# Supplementary material for: Serious Bacterial Infections in Preterm Infants: Should Their Age Be “Corrected”?
Source: J Clin Med. 2023 May 1;12(9):3242. doi: 10.3390/jcm12093242 (PMC10178985; doi:10.3390/jcm12093242)
Supplement: Supplementary file 1 [file jcm-12-03242-s001.zip › jcm-2299369-supplementary.pdf]

Table S1: Rate of serious bacterial infection in the different preterm age groups

Diagnosis	All patients (n=141)	Group 1 (n=78)	Group 2 (n=48)	Group 3 (n=15)	group 1 vs group 2 P value	group 2 vs group 3 P value	group 1 vs group 3 P value
**SBI**	15 (10.63%)	2 (2.56%)	10 (20.8%)	5 (33.3%)	<0.001	0.33	<0.001
**Bacteremia***	6 (4.3%)	0	4 (8.3%)	2 (13.3%)	0.01	0.56	0.001
**Bacterial Meningitis**	2 (1.4%)	0	2 (4.16%)	0	0.07	0.44	1
**Urinary tract infection**	12 (8.5%)	2 (2.6%)	6 (12.5%)	4 (26.7%)	0.03	0.18	0.0005

***All cases of meningitis and 3 cases of Urinary tract infection also had bacteremia.**

SBI: serious bacterial infection

Table S2: Postnatal complications during NICU admission

	All patients (n=141)	Group 1 (n=78)	Group 2 (n=48)	Group 3 (n=15)	group 1 vs group 2	group 2 vs group 3	group 1 vs group 3
**Postnatal complications**							
**EOS**	4 (2.83%)	3 (3.84%)	1 (2.08%)	0	0.85	0.58	0.45
**LOS**	36 (25.53%)	19 (24.35%)	14 (29.16%)	3 (20%)	0.8	0.49	0.73
**RDS**	76 (53.9%)	44 (56.41%)	25 (52.16%)	7 (46.7%)	0.88	0.74	0.48
BPD	46 (32.6%)	22 (28.2%)	20 (41.6%)	4 (26.7%)	0.12	0.28	0.86
**NEC**	20 (14.18%)	10 (12.82%)	9 (18.75%)	1 (6.7%)	0.69	0.26	0.51
**IVH**	31 (21.98%)	19 (24.35%)	11 (22.91%)	1 (6.7%)	0.7	0.16	0.13
**VP shunt**	12 (8.51%)	10 (12.82%)	2 (4.16%)	0	0.78	0.43	0.14
**Age (days) at discharge after birth**	80 ± 41	84 ± 48	80 ± 31	58 ± 17	0.69	0.01	0.04

EOS: early onset sepsis, LOS: late onset sepsis, RDS: respiratory distress syndrome, BPD: Bronchopulmonary dysplasia
NEC: Necrotizing enterocolitis, IVH: Intraventricular hemorrhage, VP: ventriculo-peritoneal shunt.

Table S3: Postnatal complications in preterm infants with SBI during NICU admission

Postnatal complications	All patients	Group 1	Group 2	Group 3
	(n=15)	(n=2)	(n=8)	(n=5)
**EOS**	1 (6.7%)	0	1 (12.5%)	0
**LOS**	4 (26.7%)	1 (50%)	3 (37.5%)	0
**RDS**	12 (80%)	2 (100%)	6 (75%)	4 (80%)
BPD	9 (60)	1 (50%)	4 (50%)	4 (80%)
**NEC**	4 (26.7%)	1 (50%)	3 (37.5%)	0
**IVH**	3 (20%)	1 (50%)	2 (25%)	0
**VP shunt**	2 (13.3%)	1 (50%)	1 (12.5%)	0

EOS: early onset sepsis, LOS: late onset sepsis, RDS: respiratory distress syndrome, BPD: Bronchopulmonary dysplasia, NEC: Necrotizing enterocolitis, IVH: Intraventricular hemorrhage, VP: ventriculo-peritoneal shunt.

Table S4: Ancillary laboratory test results in preterm infants with SBI

	All patients	Group 1	Group 2	Group 3	group 1 vs group 2	group 2 vs group 3	group 1 vs group 3
	(n=15)	(n=2)	(n=8)	(n=5)	P value	P value)	P value
**Fever**	12 (80%)	2 (100%)	7 (87.5%)	3 (60%)	0.61	0.27	0.32
**WBC**	12 (4.1 - 34.8)	11.65 (5.9 - 34.8)	12.65 (4.1 - 24.6)	8.7 (4.3-31.2)	0.78	0.53	0.68
**CRP**	2.1 (0.01 - 27)	1.5 (0.01 - 27)	2.96 (0.01 - 14.2)	0.1 (0.01-6)	0.35	0.57	0.33
**Positive urinalysis**	7 (46.66%)	2 (100%)	3 (37.5%)	2 (40%)	0.13	0.93	0.18
**Pleocytosis in CSF**	6 (40%)	0	2 (25%)	4 (80%)	0.45	0.06	0.07

WBC: white blood cells, CRP: C- reactive protein, CSF: Cerebrospinal fluid
